# Enhancing medical assessment strategies: a comparative study between structured, traditional and hybrid viva-voce assessment

**DOI:** 10.1186/s12909-025-07428-9

**Published:** 2025-06-04

**Authors:** Kavita Rasalkar, Sunita Tripathy, Sulekha Sinha, Bikramaditya Mukherjee, Nagamma Takkella, Elvis Vishal Dadel, Monit Sundriyal, Swetanka Prasad

**Affiliations:** Department of Biochemistry, Manipal Tata Medical College, Manipal Academy of Higher Education, Manipal, Karnataka 576 104 India

**Keywords:** Structured viva voce, Traditional viva voce, Hybrid viva-voce, Formative assessment, Summative assessment, Examiner, Evaluator, Students’ performance, Examination, Inter examiner consistency, Mitigation of bais, Medical education, Biochemistry, Oral examination, Competency, NMC, CBME 2019

## Abstract

**Background:**

Medical students’ skills and knowledge have traditionally been assessed through written and oral examinations (viva-voce). Structured viva-voce is an objective structured method to assess students orally. As an assessment tool it was used informally since 1989, however it was described separately by Oakley and Hencken in 2005. Hybrid method is a combination of both structured and traditional viva-voce methods. This study aims to assess the methods for inter-examiner consistency to minimize variations in scoring during viva-voce. It further investigates medical students’ perceptions regarding the fairness, transparency, and overall experience of the assessment formats: structured viva-voce, traditional viva-voce, and hybrid method. By examining these perspectives, the study seeks to provide insights into optimizing viva-voce methods for improved reliability and student satisfaction.

**Methods:**

Oral assessment was scheduled. Validated structured viva-voce cards, based on Bloom’s taxonomy and reviewed by the experts were prepared. Each student underwent a 5-minute structured viva using two different card sets, followed by a 5-minute traditional viva conducted by both examiners. A hybrid format included combining both methods. Feedback was obtained through a mixed questionnaire with quantitative (Likert scale) and qualitative (open-ended) items on the examination methods.

**Results and conclusion:**

The study evaluated variation in scoring between 2 examiners for three viva-voce formats: structured, traditional, and hybrid (a combination of both traditional and structured) among 151 students with 53.6% females and 46.3% males. The Wilcoxon signed rank test revealed significant inter-examiner variability in structured viva-voce and Set 2 of traditional viva-voce (*p* < 0.05), while the hybrid method showed better consistency between examiners. Pearson correlation and reliability analyses indicated that the hybrid viva-voce demonstrated higher inter-examiner consistency, correlation coefficients, and reliability (ICC and Cronbach’s α = 0.663) compared to structured and traditional formats, suggesting it may be a more effective assessment method. Feedback revealed that 56% of students preferred the hybrid format for its balance of objectivity and flexibility. While structured viva-voces excelled in fairness and coverage, traditional viva-voces were appreciated for flexibility but suffered from inconsistency. Overall, the hybrid format emerged as the effective assessment method, offering enhanced reliability and student satisfaction by addressing the shortcomings of both individual formats. These findings suggest the potential of hybrid viva-voce in fostering a consistent and comprehensive evaluation framework.

**Clinical trail number:**

Not applicable.

**Supplementary Information:**

The online version contains supplementary material available at 10.1186/s12909-025-07428-9.

## Background

Competency-Based Medical Education (CBME) implemented in Indian medical colleges since 2019 has defined core competencies, certifiable skills, and practical instructions. Three formative assessments, along with continuous internal assessments, have been recommended, but the assessment pattern is left to university discretion [1]. Universities have specified mark distribution for each component, but no specific instructions are provided to ensure uniformity in student evaluation.

The effects of assessment practices are far more potent than any other aspects of learning [2]. Assessments influence and control students more deeply than teachers often realize [3]. “Students can, with difficulty, escape from the effects of poor teaching, they cannot (by definition, if they want to graduate) escape the effects of poor assessment” [4]. As medical educators we need to confront the ways of assessments which has the potential to cause anxiety due to bias.

Skills and knowledge of students in medical college have been traditionally assessed by written and oral examinations. Oral examinations has been recorded in use since 1815 as an “examination conducted by speech” having named as viva-voce which seems to be derived from Latin “by or with living voice”. Viva-voce examinations [1] remain an integral part of assessments of students performance. It is interviewing the student by one or more examiners to assess depth and clarity of subject knowledge, along with his decision making and presentation skills [5, 6]. Oral examinations play a vital role in assessing competencies that are difficult to evaluate through written tests or Objective Structured Practical Examination (OSPE) sessions. They provide an opportunity to gauge students’ depth of understanding, communication skills, and response efficiency in real-time [7]. Viva-Voce offers better sensitivity in both positive and negative predictability of students understanding [8]. However, viva-voce assessments can be subjected to inter-examiner variation [9, 10]. Structured and standardized oral examination could reasonably prevent errors in viva-voce caused due to halo effect, lack of central tendency, leniency, students confidence, impression of previous candidates and judgement clustering around mid-ranges [8]. Structured viva-voce was used informally since 1989, however it was described separately as an assessment tool by Oakley and Hencken in 2005 [5, 9, 11–13]. Structured viva lacks open discussions and requires high planning [14]. The hybrid method includes structured and traditional viva voce together. This method attempts to overcome the disadvantages of both the methods.

This study aims to assess methods for inter-examiner consistency and variation in different viva-voce assessment methods. It further investigates medical students’ perceptions regarding the fairness, transparency, and overall experience of three assessment formats: structured viva-voce, traditional viva-voce, and a hybrid method. By examining these perspectives, the study seeks to provide insights into optimizing viva-voce methods for improved reliability and student satisfaction.

## Methods

### Ethical consideration

The study adhered to the Declaration of Helsinki. The study was approved by the Institution Ethics Committee (IEC-DHR Registration EC/NEW/INST/2022/2810) of Manipal Tata Medical College Jamshedpur. (Ethics approval number: MTMC/IEC/2024/67)

### Study design

The study was a comparative observational cross sectional study design.

### Participants

The study participants were the phase 1 medical students 2023-24 batch of National Medical Commission (NMC) approved medical college. All 151 students studying in Manipal tata medical college Jamshedpur in Ist year MBBS were enrolled in the study with 81 females and 75 male students. Overall, six competent faculty members were involved in the study. All the faculty members were serving as Assistant Professors and above as per NMC norms for Manipal Tata Medical College. Four competent Biochemistry faculty members were in charge of conducting the viva-voce examination, and the other two were planners of the study.

### Setting of the study

This study was conducted in the department of Biochemistry during the III sessional examination of Ist MBBS students as a part of formative assessments. All the Biochemistry competencies as per the NMC -CBME curriculum 2019 were covered for the assessment. The planners trained four biochemistry faculty examiners to conduct the session. Isolated viva-voce rooms were set up for four examiners, which were labeled as Examiners 1 & 2 (S1 E1 & S1E2) of set 1 and Examiners 1 & 2 (S2 E1 & S2 E2) of set 2. No mobile phones or other gadgets were allowed in the exam hall. To curb inter-student communication, viva-voce rooms were arranged in a one-way manner so that each student left the department after completing the viva-voce. Even during the session, strict discipline was maintained, and no students were allowed to communicate with any other students.

### Instrument

#### Preparation

Structured viva-voce cards were planned and specifically prepared for this study by two competent biochemistry faculty, and it was discussed and finalized with four other biochemistry faculty who were also examiners. Planners got the pattern validated by experts in biochemistry. Competent external Biochemistry experts in grade Associate Professors and above, not associated with the study, were consulted for content validation. A structured questionnaire, as outlined in Table 1, was developed using Google Forms. The experts were requested to assess the relevance of each question using a 5-point Likert scale ranging from “strongly disagree” to “strongly agree.” The Content Validity Index (CVI) was subsequently calculated, with a CVI value of ≥ 0.7 considered acceptable for inclusion.

Structured viva-voce cards were used to evaluate students’ Biochemistry knowledge. Each 10-mark card included eight questions of increasing complexity based on Bloom’s taxonomy. Domain 1 (Knowledge) and Domain 2 (Comprehension) had two 1-mark questions each, while Domain 3 (Application & Analysis) had four (Table 1). To ensure anonymity of the cards, the cards were unmarked.

The questions included were as follows: (Table 1)

**Table 1:** Blue print of questionnaire with marking scheme

Sl no	Question	Marking	Remarks	Domain	CVI
1	Reference values of TWO parameters	1mark	Clinically important parameter covered under practical	Recall	1
2	Biochemical defect of any TWO condition	1 mark	(enzyme/vitamin/mineral deficiency or excess/ genetic defect		1
3	Describe the functions of	1 mark	Any ONE biomolecule-Phosphofructokinase, Glucagon, cholesterol	Understand	1
4	Give reasons for any clinical feature of any disease	1 mark	Example: haemolysis in sickle cell anaemia		1
5	Describe the mechanism of action of any ONE drug	1 Mark	Statin, 5 fluorouracil, methotrexate	Analysis and apply	1
6	Diagnostic significance of a biochemical test	1 Mark	HbA1c, Lipase, Troponin, CKMB		1
7	Explain the biochemical causes/etiopathogenesis of any disease	2 marks	Any ONE explanation question. Cause of cataract in galactosemia, anaemia in glucose-6 phosphate deficiency		1
8	Justify	2 marks	Any ONE Justification question example-Oxidized Low-density lipoprotein is atherogenic, prolong starvation causes ketoacidosis		1

## Process

**Fig. 1:**
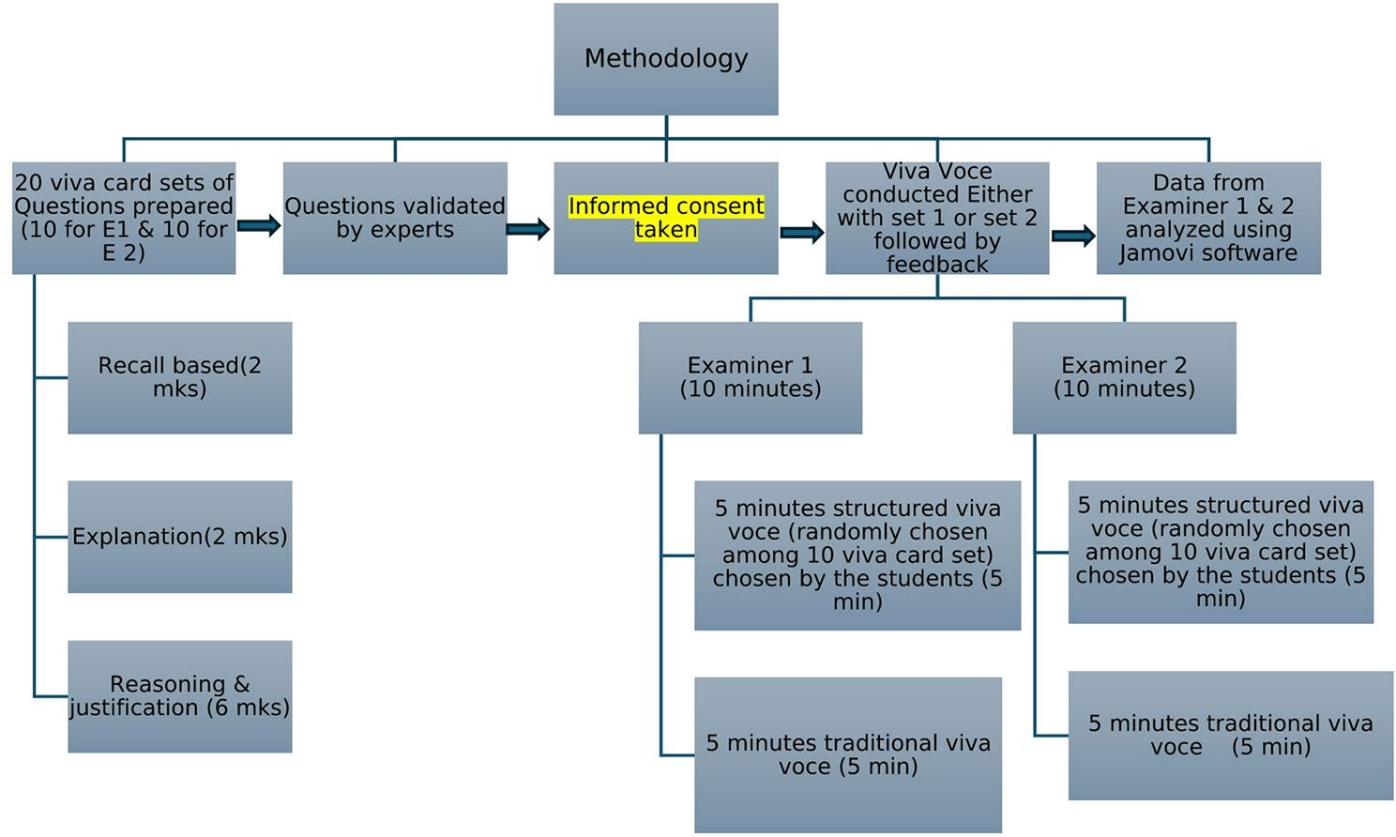
Flow chart of the methodology

The examination process was explained to the students and informed consent was obtained; all students voluntarily consented to participate in the study. Each student was instructed to complete assessments of both structured and traditional viva (5 min each = 10 min) from examiner 1 and examiner 2 sequentially. Hence, each student was assessed for 10 min with examiner 1 and 10 min with examiner 2, for a total of 20 min of viva voce. All involved faculty strictly maintained time for each session.

### Structured viva-voce session

Twenty different viva cards were prepared by planners. Each examiner received ten different viva-voce cards without any identification, kept upside down on their table. Each student randomly selected a viva-voce card by lottery system, and answered the questions mentioned in the viva-voce card in the first 5 min as a part of structured viva-voce. As per the expert’s opinion, on average the allotted time was sufficient to complete the assessment.

#### Traditional viva-voce session

In the next 5-minute session with each examiner, each student faced a traditional viva-voce examination. The faculty randomly asked questions related to the viva-voce topic without a predetermined structure and marked the students accordingly.

### Hybrid method

The marks obtained by students for structured and traditional viva-voce examinations were summed up to get the scores of the hybrid method.

#### Feedback

Following the viva-voce session, students and faculty were asked to provide feedback on their perception of the assessment methods in Microsoft forms. Quantitative feedback was obtained using Likert scaling covering aspects such as fairness, examiner consistency, student engagement, opportunity for reflection, questioning quality, content coverage and overall effectiveness with the assessment process. Qualitative feedback using open-ended questions was also gathered to gain insights into their experiences. The qualitative feedback was analyzed using content analysis to identify key themes. Responses were coded based on frequently mentioned words and recurring ideas. The primary categories included advantages and disadvantages of assessment methods.

### Statistical analysis

Descriptive statistics and a comparison of means between structured, traditional, and hybrid viva-voce methods between examiner 1 and examiner 2 were done. Since the data was skewed, the Wilcoxon signed-rank test was used to analyze paired differences in scores given by two different examiners to the same student. Intraclass Correlation Coefficient (ICC) & Cronbach α [10] was done to evaluate the reliability of measurements. Internal consistency of each viva format was assessed using Cronbach’s alpha across cases. Inter-examiner agreement was evaluated using the intraclass correlation coefficient (ICC). It was calculated based on scores independently assigned by two examiners to the same group of students across the three viva formats. Although raters did not score students simultaneously, ICC was used to provide an estimate of the consistency in scoring patterns between the two raters for each format. Linear regression to explore relationships between variables and Pearson correlation to assess the strength and direction of associations. Quantitative feedback utilizing Likert scales was done to gauge participant responses. The p value of < 0.01 was considered as statistically significant. Jamovi 2022 version 2.3 was used for statistical analysis [15]. The content analysis method was adopted for qualitative data obtained in feedback. Coding was performed manually by grouping similar responses, ensuring clarity and consistency in interpretation. This allowed for a structured analysis of perceptions regarding the effectiveness, fairness, and limitations of each viva format.

## Results

**Table 2:** Demographic results

	Examiners Set 1 *N* (%)	Examiner Set 2 *N* (%)	Total (*N*)
Females	41 (27.1%)	40 (26.5%)	81(53.6%)
Males	34 (22.5%)	36(23.8%)	70(46.3%)
Total	75 (49.7%)	76 (50.3%)	151

N = number of students

A total of 151 students participated in the study, comprising 81 females and 70 males. Male and female students were distributed approximately equally among the four examiners to ensure balanced representation (Table 2).

### Quantitative results

**Table 3:** Descriptive scores among examiner 1 and examiner 2 using the different evaluation methods

Viva-voce Type	Examiner	*N*	Min	Max	Mean *±* SD	Statistics	*P* value by paired t test	*P* value by Wilcoxon Signed rank test
Structured	S1E1	75	0	9	6.29 *±* 1.66	2.3	0.032	0.034*
	S1E2	76	2	9	5.96 *±* 1.27			
	S2E1	75	2.5	9	5.95 *±* 1.39	1.79	0.077	0.067
	S2E2	76	1.5	9.5	6.74 *±* 1.98			
	E1	151	0	9	6.26 *±* 1.97	2.83	0.005*	0.005*
	E2	151	1.5	9.5	5.78 *±* 1.92			
Traditional	S1E1	75	0	10	6.12 *±* 2.17	1.79	0.219	0.047*
	S1E2	76	2	10	5.55 *±* 1.6			
	S2E1	75	2	9	6.39 *±* 1.75	-3.89	< 0.001*	< 0.001**
	S2E2	76	1.5	10	6 *±* 2.19			
	E1	151	0	10	6.12 *±* 1.54	-1.54	0.125	0.220
	E2	151	1.5	10	6.35 *±* 1.7			
Hybrid	S1E1	75	0	19	12.3 *±* 3.68	2.5	0.016	0.002*
	S1E2	76	4.5	19	11.5 *±* 2.62			
	S2E1	75	6	17	12.3 *±* 2.91	-1.34	0.182	0.3
	S2E2	76	4	19.5	12.7 *±* 3.6			
	E1	151	0	19	12.3 *±* 3.31	0.646	0.519	0.213
	E2	151	4	19.5	12.1 *±* 3.2			

SD: standard deviation; Min: minimum value; Max: maximum value; S1E1: Set 1 Examiner 1; S1E2: Set 1 Examiner 2; S2E1: Set 2 Examiner 1; S2E2: Set 2 Examiner 2. P: statistical significance between examiners (*p* < 0.01)

Table 3 presents the descriptive statistics for student scores awarded by Examiners 1 and 2 across three assessment formats: structured viva-voce, traditional viva-voce, and the hybrid method. Both the structured and traditional viva-voce formats exhibited inter-examiner variation with the Wilcoxon signed rank test. A p-value of < 0.05 was considered statistically significant and < 0.01 as highly statistically significant. For set 1 and overall, of structured viva-voce, Set 1& 2 of traditional viva-voce, set 1 of hybrid method the p-value was statistically significant, indicating notable inter-examiner variability in these formats. Traditional viva showed a highly statistically significant difference of < 0.001.

**Table 4:** Linear regression

Viva-voce Type	Examiner	*R* value	R2	Estm	t Value	*P* value
Structured	Set 1	0.148	0.021	-0.566	-1.83	0.069
	Set 2	0.098	0.0099	-0.395	-1.23	0.221
	Together	0.424	0.180	0.434	5.73	< 0.001*
Traditional	Set 1	0.11	0.122	-0.36	-1.36	0.177
	Set 2	0.225	0.0504	0.784	2.81	0.006*
	Together	0.458	0.210	0.413	6.26	< 0.001*
Hybrid	Set 1	0.127	0.016	-0.809	-1.56	0.120
	Set 2	0.0717	0.00513	0.467	0.880	0.380
	Together	0.496	0.246	0.513	6.99	< 0.001*

R value: correlation coefficient, R2: Estm: Estimate (regression coefficient), t-Value: test statistic value P: statistical significance between examiners (*p* < 0.01)

Table 4 illustrates the findings of linear regression analysis performed on the scores assigned by Examiners 1 and 2 for structured, traditional, and hybrid viva-voce formats. Statistically significant associations were observed between the examiners’ scores across all three formats. The correlation coefficients indicated moderate correlation, with values of 0.424 for structured viva-voce and 0.458 for traditional viva-voce. The strongest association was observed in hybrid mode, with a correlation coefficient of 0.496. This suggests that the hybrid format facilitates better alignment between examiners’ scorings. While structured viva-voce allows for a more detailed evaluation, inter-examiner variability persists.

**Table 5:** Correlation matrix and reliability analysis

		Pearsons Correlation		Cronbach’s α	ICC
Viva-voce Type	Examiner	R value	P value		
Structured	Set 1	0.159	0.170	0.460	0.442
	Set 2	0.545	< 0.001	0.694	0.646
	Together	0.424	< 0.001	0.595	0.511
Traditional	Set 1	0.421	< 0.001	0.568	0.542
	Set 2	0.537	< 0.001	0.671	0.576
	Together	0.458	< 0.001	0.626	0.551
Hybrid	Set 1	0.393	< 0.001	0.602	0.551
	Set 2	0.587	< 0.001	0.729*	0.685
	Together	0.496	< 0.001	0.663	0.591

R value: correlation coefficient, P: statistical significance between examiners (*p* < 0.01)

Pearson correlation analyses were performed to further assess the relationship between scores assigned by Examiners 1 and 2 across the three viva-voce formats (Table 5). Statistically significant associations were observed for the structured, traditional, and hybrid modes, except set 1 of structured viva-voce. Internal consistency, as an indicator of reliability, was measured using Cronbach’s alpha. A Cronbach alpha value greater than 0.7 is acceptable, greater than 0.8 is considered good and above 0.9 is excellent reliability [10, 16]. Reliability analysis using Cronbach’s α in this study showed low reliability of 0.595 for structured viva-voce and moderate reliability for traditional (0.626) and hybrid viva-voce method (0.663) (Table 5). Only hybrid viva-voce Cronbach α reached the threshold value of > 0.7. In comparison, the hybrid viva-voce consistently demonstrated higher correlation coefficients and reliability than the structured and traditional formats, suggesting that it may be a more effective assessment method or that examiners’ grading aligns more closely in this format. One-way random interclass correlation coefficient (ICC) (< 0.5 poor, 0.5–0.75 moderate, > 0.75 good reliability) was slightly higher for hybrid when compared to structured or traditional viva-voce, indicating higher inter rater(inter-examiner) consistency and reliability (Table 5). These findings highlight the potential of the hybrid viva voce in reducing inter-examiner variability while ensuring consistent and comprehensive evaluation.

### Feedback analysis

**Fig. 2:**
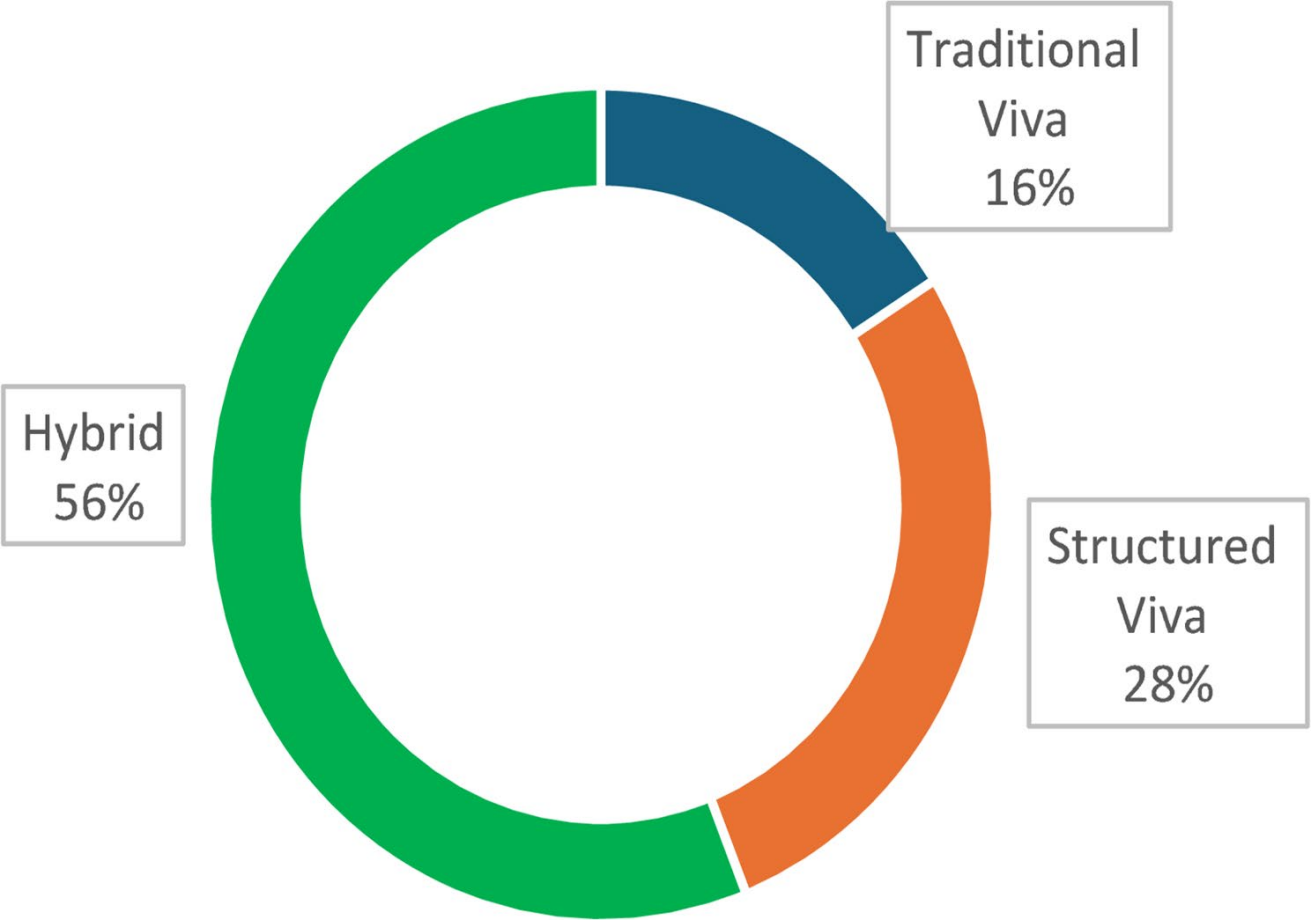
Students preference percentages for different viva-voce methods

**Fig. 3:**
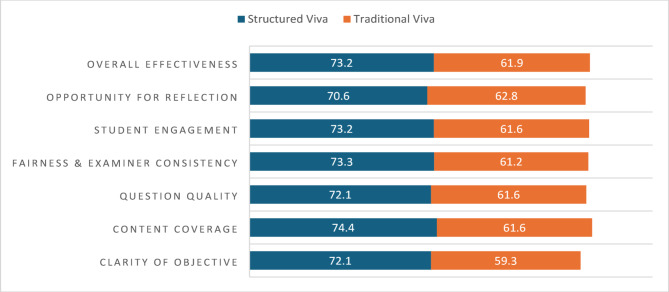
Quantitative feedback analysis

The students’ feedback was taken as the quantitative Likert scaling and open-ended qualitative questions. In the study, 16% of students expressed a preference for the traditional viva-voce format, while 28% favored the structured viva-voce and the majority of students (56%) preferred a hybrid approach that combined elements of both assessment methods (Fig. 2). Quantitative feedback was collected using a Likert scale to evaluate structured and traditional viva-voce formats across various parameters. A significantly higher percentage of students rated the structured viva voce positively compared to the traditional viva voce, particularly in aspects such as content coverage and clarity of objectives (Fig. 3).

**Table 6:** Qualitative feedback analysis

	Strengths	Limitations
Structured viva-voce	**Objectivity and fairness**: • Helps consistent and unbiased marking. • Consistency of questions helps students to prepare and perform better. • Questions from all topics, hence better coverage of topics. • Consistency on questioning by all examiners Helps build **student confidence** by focusing on well-defined topics Completes in equal time frame for all	Higher preparation time for faculty **Rigid & less flexible**: • Low scope for exploring topics beyond the listed questions • Unprepared students lose marks easily • Provides efficacy but is not ideal for assessing deeper understanding
Traditional viva-voce	**Flexibility**: • Encourages underprepared students to answer questions • Encourages prepared students to assess depth of Knowledge; broader preparation is encouraged. Lower chances of cheating Completes in a **shorter time** frame	Unpredictable hence causes anxiety among students **Examiner variability**: • Inconsistency in covering broader range of topics • Students worry about examiner-specific questions (Pet Questions) Option to change questions can cause an **uneven** assessment process Lack of structure causes **unfairness**

Qualitative feedback was also analyzed from open-ended questions regarding the perceived advantages and disadvantages of both formats. Students highlighted that the structured viva voce’s primary advantages were its objectivity and fairness, although its rigidity was identified as a drawback. Conversely, the traditional viva-voce was appreciated for its flexibility, but students noted examiner-to-examiner variability as a significant disadvantage (Table 6). The feedback from the faculty revealed the strengths of the method, as consistency in questioning patterns, and limitations, such as the higher preparation time for conducting the assessment sessions. Fairness was the most common word used for structured viva and flexibility was the most common word for traditional viva.

## Discussion

In this study, viva-voce assessment methods were analyzed to evaluate inter-examiner consistency, variation, and check transparency and fairness in the scoring system. The qualitative and quantitative data obtained from the study were complementary to each other. Quantitative data were analyzed through descriptive statistics, correlation analysis, reliability analysis, and Likert scale for feedback, while qualitative insights were gathered through open-ended feedback questions from students and faculty.

Structured viva-voce, as supported by examiners in this study and consistent with findings by Gor et al. [17], has the potential to reduce scoring bias through carefully selecting questions targeting the cognitive domains of Bloom’s taxonomy, including knowledge, comprehension, application, and analysis. However, our findings revealed similar mean values as seen in study by Shaikh et al. [18], low consistency in scoring between examiners for both structured and traditional viva-voce, with statistically significant differences in scores across different sets of examiners. These results align with those reported by Khilnani et al. [19]. In contrast, the hybrid mode of assessment demonstrated statistically significant differences in scoring between two examiners evaluating the same student only in set 1, highlighting its potential for improved consistency. However, the reduction in variation and improved reliability due to averaging the scores for both structured and traditional assessments cannot be overlooked. Further studies involving the randomization of students into different groups and a detailed comparison of scoring differences between examiners could provide clearer insights.

As observed by Gor et al. [17], correlation analysis in this study showed statistically significant associations across all three assessment methods: structured, traditional, and hybrid modes. However, unlike the findings of Suwarna et al. [16, 20], reliability analysis in our study showed low reliability, primarily attributed to scoring variations with set 1 examiners. While our study focuses on psychometric indicators such as Cronbach’s alpha and ICC to assess interrater reliability, we acknowledge that validity encompasses more than statistical consistency. Addressing the variations through intensive examiner training in assessment strategies, calibration sessions to standardize questions, along with mock practice sessions for students could enhance reliability and effectiveness in the future as suggested by Kane [6, 17, 21] The absence of examiner scripting by design in traditional viva is a known threat to scoring and generalization validity. In contrast, structured and hybrid formats, by using prompts and rubrics, better support consistent interpretation and use of scores.

Quantitative feedback analysis using a Likert scale demonstrated a clear preference for structured viva-voce and the hybrid mode over the traditional assessment method. Structured viva-voce received favorable ratings across all measured parameters compared to traditional viva-voce, aligning with findings from Shenwai et al. [22]. Content analysis for qualitative feedback data revealed distinct perceptions of traditional and structured viva-voce methods among students and faculty. Students viewed the traditional viva-voce as flexible, with lower chances of cheating, and quicker to complete. However, they highlighted significant drawbacks, including unpredictability, higher examiner variability, and pressure to anticipate faculty-specific “pet” questions. The option for examiners to change questions at a student’s request was noted to have the potential for biased marking and unfair assessments. In contrast, the structured viva-voce was appreciated for its objectivity, fairness, predictability, and focus on well-defined topics, enabling students to prepare effectively and achieve higher satisfaction due to broader topic coverage. However, students noted its rigidity, which could be discouraging for weaker candidates, and its inability to assess deeper understanding. “Equality for all students irrespective of any teacher” “No feeling of only certain specific questions to be asked by each of the faculties.” Were few of the comments regarding structured viva & “Teacher can decide what level of questions to ask from whom” “Low chances of cheating” “Flexibility” were the comments for traditional viva.

The examiners opined that structured viva voce required more preparation time, which could be a deterrent. Nevertheless, they emphasized that if standardized could enable unbiased, systematic assessment fostering critical evaluation of students’ knowledge and skills.

To the best of our knowledge, no prior studies have specifically evaluated the effectiveness of the hybrid mode of assessment. Our findings reveal that the hybrid mode offers statistically significant advantages, including superior inter-examiner consistency, reduced bias, and better correlation. Notably, it emerged as the preferred assessment method among students. This innovative approach combines the structured evaluation of a standardized viva with the flexibility of the traditional method, ensuring unbiased assessments while allowing for a deeper analysis of students’ understanding and covering a broader range of topics. Additionally, the hybrid mode mitigates anxiety and fear associated with examiner-related variations in scoring by fostering transparency and fairness. Its ability to blend objectivity with adaptability makes it a promising model for comprehensive and equitable evaluation in medical education.

### Limitations of the study

The study’s findings may be influenced by the single centric, specific context and the chosen subject, limiting their generalizability to all medical education settings. Additionally, the longer preparation time required for faculty may restrict the widespread adoption of this method. However, once established, the standardized structured viva material can be utilized consistently in subsequent assessments.

Students underwent assessments by two different examiners, thereby prolonging their evaluation time. Since neither randomization nor a crossover design was implemented, and both viva techniques were conducted in quick succession, faculty scoring may have been influenced by prior responses. Faculty training for the structured viva may have influenced the traditional viva results, affecting comparison. Two examiners observing and scoring a single examination process could have given a better understanding of interrater consistency. While the hybrid mode appeared to be well-received by advanced learners—allowing them to answer structured viva questions rapidly while elaborating on concepts in the traditional viva—the observed statistical significance may have been influenced by the larger sample size rather than a true advantage of the method.

Faculty feedback was collected through Microsoft forms, but better documentation could be achieved through recorded interview sessions for in-depth discussion. Lastly, improving the design of viva cards and providing intensive examiner training could enhance consistency and reliability in scoring across different evaluators.

## Conclusion

Overall, the hybrid format emerged as the most effective assessment method, offering enhanced reliability and student satisfaction by addressing the shortcomings of both individual formats. These findings suggest the potential of hybrid viva-voce in fostering a consistent and comprehensive evaluation framework. It can be implemented in medical colleges by integrating structured with traditional assessments, ensuring standardization, reducing examiner variation, and enhancing comprehensive evaluation of students’ clinical reasoning and knowledge. The well-structured questionnaire can improve inter-rater reliability. Further studies are warranted to support the hybrid model as an effective and reliable viva-voce assessment method. We shall plan to collaborate with other institutions to adopt a multicentric and multidisciplinary approach.”

## Electronic supplementary material

Below is the link to the electronic supplementary material.

Supplementary Material 1

Supplementary Material 2

Supplementary Material 3

Supplementary Material 4

## Data Availability

Raw de-identified data may be made available upon reasonable request to the corresponding authors.

## References

[1] UG-Curriculum-Vol. -I.pdf [Internet]. [cited 2024 Jul 27]. Available from: https://www.nmc.org.in/wp-content/uploads/2020/01/UG-Curriculum-Vol-I.pdf

[2] Basheer A. Impact of assessment of medical students in India on assuring quality primary care. Australas Med J. 2015;8(2):67–9. 25810790 10.4066/AMJ.2015.2297 PMC4354027

[3] Epstein RM. Assessment in medical education. Cox M, Irby DM, editors. N Engl J Med. 2007;356(4):387–96. 10.1056/NEJMra054784 17251535

[4] Boud D. Assessment and learning: contradictory or complementary?? Assessment for learning in higher education. Routledge; 1995.

[5] Dhasmana DC, Bala S, Sharma R, Sharma T, Kohli S, Aggarwal N, et al. Introducing structured Viva Voce examination in medical undergraduate pharmacology: A pilot study. Indian J Pharmacol. 2016;48(Suppl 1):S52–6. 28031609 10.4103/0253-7613.193308 PMC5178057

[6] Anbarasi K, Karunakaran J, Ravichandran L, Arthi B. effectiveness of the structured and conventional methods of viva examination in medical education: a systematic review and meta-analysis. J Clin Diagn Res [Internet]. 2022 [cited 2024 Jul 27]; Available from: https://www.jcdr.net//article_fulltext.asp?issn=0973-709x%26year=2022%26volume=16%26issue=9%26page=JE01%26issn=0973-709x%26id=16977

[7] Anurag Agarwal B, Batra AK, Sood. Evolutionary trends in radiology assessment: The importance of the learning cycle and its assessment in radiology. 10.4103/0971-3026.43833 PMC2747460 19774177

[8] de Silva V, Hanwella R, Ponnamperuma G. The validity of oral assessment (viva) that assesses specific and unique competencies in a post-graduate psychiatry examination. Sri Lanka J Psychiatry [Internet]. 2013 Jan 13 [cited 2024 Dec 16];3(2). Available from: https://sljpsyc.sljol.info/articles/10.4038/sljpsyc.v3i2.5133

[9] Imran M, Doshi C, Kharadi D. Structured and unstructured viva voce assessment: a double-blind, randomized, comparative evaluation of medical students. Int J Health Sci. 2019;13(2). PMC6436443 30983939

[10] Abuzied AIH, Nabag WOM. Structured Viva validity, reliability, and acceptability as an assessment tool in health professions education: a systematic review and meta-analysis. BMC Med Educ. 2023;23(1):531. 37491301 10.1186/s12909-023-04524-6 PMC10369684

[11] Tutton PJM, Glasgow EF. Reliability and predictive capacity of examinations in anatomy and improvement in the reliability of Viva Voce (oral) examinations by the use of a structured rating system. Clin Anat. 1989;2(1):29–34.

[12] Thomas CS, Mellsop G, Callender K, Crawshaw J, Ellis PM, Hall A, et al. The oral examination: a study of academic and non-academic factors. Med Educ. 1993;27(5):433–9. 8208147 10.1111/j.1365-2923.1993.tb00297.x

[13] Oakley B, Hencken C. Oral examination assessment practices: effectiveness and change within a first year undergraduate cohort. J Hosp Leis Sport Tour Educ. 2005;4(1):3–14.

[14] Ahsan M, Mallick A. A study to assess the reliability of structured Viva examination over traditional Viva examination among 2nd–Year Pharmacology students. J Datta Meghe Inst Med Sci Univ. 2022;17:589–94.

[15] jamovi - open. statistical software for the desktop and cloud [Internet]. [cited 2024 Apr 17]. Available from: https://www.jamovi.org/

[16] Suwarna Madhukumar, Pavithra MB. Conventional Viva and structured Viva — Comparison and perception of students. Indian J Public Health Res Dev. 2022;13(2):167–72.

[17] Gor S, Budh D, Athanikar B. Comparison of conventional Viva examination with objective structured Viva in second year pathology students. Int J Med Sci Public Health. 2014;3(5):537.

[18] Shaikh ST. Objective Structured viva examination versus traditional viva examination in evaluation of medical students. Anat Physiol [Internet]. 2015 [cited 2024 Jul 27];05(03). Available from: https://www.omicsonline.org/open-access/objective-structured-viva-examination-versus-traditional-viva-examination-inevaluation-of-medical-students-2161-0940-1000175.php?aid=53387

[19] Khilnani AK, Charan J, Thaddanee R, Pathak RR, Makwana S, Khilnani G. Structured oral examination in Pharmacology for undergraduate medical students: factors influencing its implementation. Indian J Pharmacol. 2015;47(5):546. 26600646 10.4103/0253-7613.165182 PMC4621678

[20] Ganji KK. Evaluation of reliability in structured *Viva Voce* as a formative assessment of dental students. J Dent Educ. 2017;81(5):590–6. 28461636 10.21815/JDE.016.017

[21] Sireci S. On validity theory and test validation. Educ Res. 2007;36.

[22] Shenwai MR, Patil B. Introduction of structured oral examination as A novel assessment tool to first year medical students in physiology. J Clin Diagn Res JCDR. 2013;7(11):2544–7. 24392396 10.7860/JCDR/2013/7350.3606 PMC3879849

