# Supplementary material for: Enhancing medical assessment strategies: a comparative study between structured, traditional and hybrid viva-voce assessment
Source: BMC Med Educ. 2025 Jun 4;25:835. doi: 10.1186/s12909-025-07428-9 (PMC12139079; doi:10.1186/s12909-025-07428-9)
Supplement: Supplementary file 2 — Supplementary Material 2 [file 12909_2025_7428_MOESM2_ESM.docx]

**Correlation Matrix**

| Correlation Matrix | | | | | | | | | | | | | | | |
| --- | --- | --- | --- | --- | --- | --- | --- | --- | --- | --- | --- | --- | --- | --- | --- |
|  | |  | | **S2 E1 SV** | | **S2 E1 TV** | | **S2 E2 SV** | | **S2 E2 TV** | | **S2 Hybrid E1** | | **S2 Hybrid E2** | |
| S2 E1 SV |  | Pearson's r |  | — |  |  |  |  |  |  |  |  |  |  |  |
|  |  | df |  | — |  |  |  |  |  |  |  |  |  |  |  |
|  |  | p-value |  | — |  |  |  |  |  |  |  |  |  |  |  |
|  |  | Spearman's rho |  | — |  |  |  |  |  |  |  |  |  |  |  |
|  |  | df |  | — |  |  |  |  |  |  |  |  |  |  |  |
|  |  | p-value |  | — |  |  |  |  |  |  |  |  |  |  |  |
| S2 E1 TV |  | Pearson's r |  | 0.615 |  | — |  |  |  |  |  |  |  |  |  |
|  |  | df |  | 73 |  | — |  |  |  |  |  |  |  |  |  |
|  |  | p-value |  | < .001 |  | — |  |  |  |  |  |  |  |  |  |
|  |  | Spearman's rho |  | 0.607 |  | — |  |  |  |  |  |  |  |  |  |
|  |  | df |  | 73 |  | — |  |  |  |  |  |  |  |  |  |
|  |  | p-value |  | < .001 |  | — |  |  |  |  |  |  |  |  |  |
| S2 E2 SV |  | Pearson's r |  | 0.545 |  | 0.513 |  | — |  |  |  |  |  |  |  |
|  |  | df |  | 74 |  | 73 |  | — |  |  |  |  |  |  |  |
|  |  | p-value |  | < .001 |  | < .001 |  | — |  |  |  |  |  |  |  |
|  |  | Spearman's rho |  | 0.584 |  | 0.501 |  | — |  |  |  |  |  |  |  |
|  |  | df |  | 74 |  | 73 |  | — |  |  |  |  |  |  |  |
|  |  | p-value |  | < .001 |  | < .001 |  | — |  |  |  |  |  |  |  |
| S2 E2 TV |  | Pearson's r |  | 0.405 |  | 0.537 |  | 0.492 |  | — |  |  |  |  |  |
|  |  | df |  | 74 |  | 73 |  | 74 |  | — |  |  |  |  |  |
|  |  | p-value |  | < .001 |  | < .001 |  | < .001 |  | — |  |  |  |  |  |
|  |  | Spearman's rho |  | 0.402 |  | 0.508 |  | 0.469 |  | — |  |  |  |  |  |
|  |  | df |  | 74 |  | 73 |  | 74 |  | — |  |  |  |  |  |
|  |  | p-value |  | < .001 |  | < .001 |  | < .001 |  | — |  |  |  |  |  |
| S2 Hybrid E1 |  | Pearson's r |  | 0.899 |  | 0.872 |  | 0.531 |  | 0.481 |  | — |  |  |  |
|  |  | df |  | 74 |  | 73 |  | 74 |  | 74 |  | — |  |  |  |
|  |  | p-value |  | < .001 |  | < .001 |  | < .001 |  | < .001 |  | — |  |  |  |
|  |  | Spearman's rho |  | 0.900 |  | 0.873 |  | 0.555 |  | 0.471 |  | — |  |  |  |
|  |  | df |  | 74 |  | 73 |  | 74 |  | 74 |  | — |  |  |  |
|  |  | p-value |  | < .001 |  | < .001 |  | < .001 |  | < .001 |  | — |  |  |  |
| S2 Hybrid E2 |  | Pearson's r |  | 0.554 |  | 0.608 |  | 0.879 |  | 0.848 |  | 0.587 |  | — |  |
|  |  | df |  | 74 |  | 73 |  | 74 |  | 74 |  | 74 |  | — |  |
|  |  | p-value |  | < .001 |  | < .001 |  | < .001 |  | < .001 |  | < .001 |  | — |  |
|  |  | Spearman's rho |  | 0.581 |  | 0.582 |  | 0.877 |  | 0.827 |  | 0.598 |  | — |  |
|  |  | df |  | 74 |  | 73 |  | 74 |  | 74 |  | 74 |  | — |  |
|  |  | p-value |  | < .001 |  | < .001 |  | < .001 |  | < .001 |  | < .001 |  | — |  |
|  | | | | | | | | | | | | | | | |

| Reliability analysis  Scale Reliability Statistics | | | | | | | | | |
| --- | --- | --- | --- | --- | --- | --- | --- | --- | --- |
|  | | **Cronbach's α** | | | | **McDonald's ω** | | | |
| scale |  | 0.595 | | |  | 0.596 | | |  |
|  | | | | | | | | | |
| Scale Reliability Statistics | | | | | | | |  |  |
|  | | | | **Cronbach's α** | | | |  |  |
| scale | | |  | 0.626 | | |  |  |  |
|  | | | | | | | |  |  |

| Scale Reliability Statistics | | | |
| --- | --- | --- | --- |
|  | | **Cronbach's α** | |
| scale |  | 0.663 |  |
|  | | | |

| Scale Reliability Statistics | | | |
| --- | --- | --- | --- |
|  | | **Cronbach's α** | |
| scale |  | 0.694 |  |
|  | | | |

| Scale Reliability Statistics | | | |
| --- | --- | --- | --- |
|  | | **Cronbach's α** | |
| scale |  | 0.460 |  |
|  | | | |

| Scale Reliability Statistics | | | |
| --- | --- | --- | --- |
|  | | **Cronbach's α** | |
| scale |  | 0.671 |  |
|  | | | |

| Scale Reliability Statistics | | | |
| --- | --- | --- | --- |
|  | | **Cronbach's α** | |
| scale |  | 0.568 |  |
|  | | | |

| Scale Reliability Statistics | | | |
| --- | --- | --- | --- |
|  | | **Cronbach's α** | |
| scale |  | 0.602 |  |
|  | | | |

| Scale Reliability Statistics | | | |
| --- | --- | --- | --- |
|  | | **Cronbach's α** | |
| scale |  | 0.729 |  |
|  | | | |

Reliability analysis:

**Reliability Analysis**

Coefficient of Variation (%): 21.87Standard Error of Measurement (SEM): 1.3556Standard Error of the Estimate (SEE): 1.4045Standard Error of Prediction (SEP): 2.3843

| Intraclass Correlation Coefficients | | | | | | | | | | | |
| --- | --- | --- | --- | --- | --- | --- | --- | --- | --- | --- | --- |
| **Model** | | **Measures** | | **Type** | | **ICC** | | **Lower C.I.** | | **Upper C.I.** | |
| one-way random |  | Agreement |  | ICC1 |  | 0.521 |  | 0.369 |  | 0.646 |  |
| two-way random |  | Agreement |  | ICC2 |  | 0.524 |  | 0.374 |  | 0.648 |  |
| two-way fixed |  | Consistency |  | ICC3 |  | 0.531 |  | 0.381 |  | 0.654 |  |
| one-way random |  | Avg. Agreement |  | ICC1k |  | 0.685 |  | 0.539 |  | 0.785 |  |
| two-way random |  | Avg. Agreement |  | ICC2k |  | 0.688 |  | 0.544 |  | 0.786 |  |
| two-way fixed |  | Avg. Consistency |  | ICC3k |  | 0.694 |  | 0.551 |  | 0.791 |  |
|  | | | | | | | | | | | |

**Plot Reliability Data**


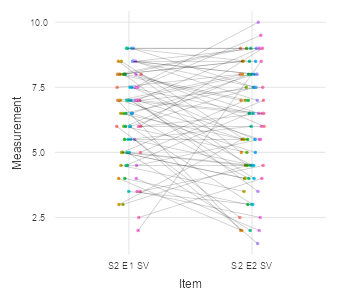


**Reliability Analysis**

Coefficient of Variation (%): 18.98Standard Error of Measurement (SEM): 1.2047Standard Error of the Estimate (SEE): 1.2406Standard Error of Prediction (SEP): 2.143

| Intraclass Correlation Coefficients | | | | | | | | | | | |
| --- | --- | --- | --- | --- | --- | --- | --- | --- | --- | --- | --- |
| **Model** | | **Measures** | | **Type** | | **ICC** | | **Lower C.I.** | | **Upper C.I.** | |
| one-way random |  | Agreement |  | ICC1 |  | 0.435 |  | 0.269 |  | 0.576 |  |
| two-way random |  | Agreement |  | ICC2 |  | 0.460 |  | 0.273 |  | 0.607 |  |
| two-way fixed |  | Consistency |  | ICC3 |  | 0.504 |  | 0.348 |  | 0.633 |  |
| one-way random |  | Avg. Agreement |  | ICC1k |  | 0.606 |  | 0.424 |  | 0.731 |  |
| two-way random |  | Avg. Agreement |  | ICC2k |  | 0.630 |  | 0.429 |  | 0.756 |  |
| two-way fixed |  | Avg. Consistency |  | ICC3k |  | 0.670 |  | 0.517 |  | 0.775 |  |
|  | | | | | | | | | | | |

**Plot Reliability Data**


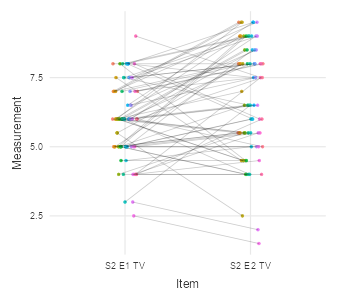


**Reliability Analysis**

Coefficient of Variation (%): 27.35Standard Error of Measurement (SEM): 1.5962Standard Error of the Estimate (SEE): 1.2481Standard Error of Prediction (SEP): 2.6014

| Intraclass Correlation Coefficients | | | | | | | | | | | |
| --- | --- | --- | --- | --- | --- | --- | --- | --- | --- | --- | --- |
| **Model** | | **Measures** | | **Type** | | **ICC** | | **Lower C.I.** | | **Upper C.I.** | |
| one-way random |  | Agreement |  | ICC1 |  | 0.277 |  | 0.0936 |  | 0.442 |  |
| two-way random |  | Agreement |  | ICC2 |  | 0.289 |  | 0.1118 |  | 0.450 |  |
| two-way fixed |  | Consistency |  | ICC3 |  | 0.299 |  | 0.1168 |  | 0.462 |  |
| one-way random |  | Avg. Agreement |  | ICC1k |  | 0.434 |  | 0.1712 |  | 0.613 |  |
| two-way random |  | Avg. Agreement |  | ICC2k |  | 0.448 |  | 0.2010 |  | 0.621 |  |
| two-way fixed |  | Avg. Consistency |  | ICC3k |  | 0.460 |  | 0.2092 |  | 0.632 |  |
|  | | | | | | | | | | | |

**Plot Reliability Data**


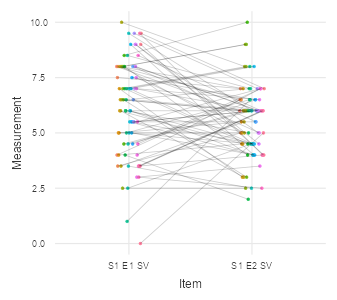


**Reliability Analysis**

Coefficient of Variation (%): 18.98Standard Error of Measurement (SEM): 1.2047Standard Error of the Estimate (SEE): 1.2406Standard Error of Prediction (SEP): 2.143

| Intraclass Correlation Coefficients | | | | | | | | | | | |
| --- | --- | --- | --- | --- | --- | --- | --- | --- | --- | --- | --- |
| **Model** | | **Measures** | | **Type** | | **ICC** | | **Lower C.I.** | | **Upper C.I.** | |
| one-way random |  | Agreement |  | ICC1 |  | 0.435 |  | 0.269 |  | 0.576 |  |
| two-way random |  | Agreement |  | ICC2 |  | 0.460 |  | 0.273 |  | 0.607 |  |
| two-way fixed |  | Consistency |  | ICC3 |  | 0.504 |  | 0.348 |  | 0.633 |  |
| one-way random |  | Avg. Agreement |  | ICC1k |  | 0.606 |  | 0.424 |  | 0.731 |  |
| two-way random |  | Avg. Agreement |  | ICC2k |  | 0.630 |  | 0.429 |  | 0.756 |  |
| two-way fixed |  | Avg. Consistency |  | ICC3k |  | 0.670 |  | 0.517 |  | 0.775 |  |
|  | | | | | | | | | | | |

**Plot Reliability Data**


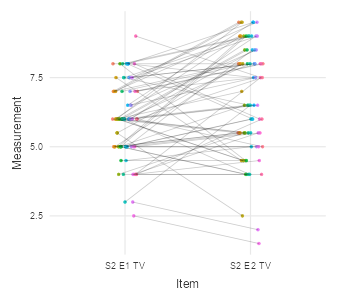


**Reliability Analysis**

Coefficient of Variation (%): 19.9Standard Error of Measurement (SEM): 1.2104Standard Error of the Estimate (SEE): 1.0808Standard Error of Prediction (SEP): 2.0289

| Intraclass Correlation Coefficients | | | | | | | | | | | |
| --- | --- | --- | --- | --- | --- | --- | --- | --- | --- | --- | --- |
| **Model** | | **Measures** | | **Type** | | **ICC** | | **Lower C.I.** | | **Upper C.I.** | |
| one-way random |  | Agreement |  | ICC1 |  | 0.393 |  | 0.222 |  | 0.542 |  |
| two-way random |  | Agreement |  | ICC2 |  | 0.395 |  | 0.224 |  | 0.542 |  |
| two-way fixed |  | Consistency |  | ICC3 |  | 0.396 |  | 0.224 |  | 0.544 |  |
| one-way random |  | Avg. Agreement |  | ICC1k |  | 0.565 |  | 0.363 |  | 0.703 |  |
| two-way random |  | Avg. Agreement |  | ICC2k |  | 0.566 |  | 0.366 |  | 0.703 |  |
| two-way fixed |  | Avg. Consistency |  | ICC3k |  | 0.568 |  | 0.366 |  | 0.705 |  |
|  | | | | | | | | | | | |

**Plot Reliability Data**


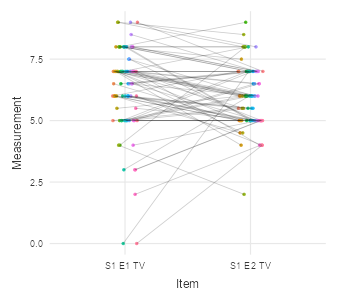


**Reliability Analysis**

Coefficient of Variation (%): 19.86Standard Error of Measurement (SEM): 2.3809Standard Error of the Estimate (SEE): 2.2351Standard Error of Prediction (SEP): 4.0733

| Intraclass Correlation Coefficients | | | | | | | | | | | |
| --- | --- | --- | --- | --- | --- | --- | --- | --- | --- | --- | --- |
| **Model** | | **Measures** | | **Type** | | **ICC** | | **Lower C.I.** | | **Upper C.I.** | |
| one-way random |  | Agreement |  | ICC1 |  | 0.404 |  | 0.234 |  | 0.551 |  |
| two-way random |  | Agreement |  | ICC2 |  | 0.415 |  | 0.248 |  | 0.559 |  |
| two-way fixed |  | Consistency |  | ICC3 |  | 0.431 |  | 0.263 |  | 0.573 |  |
| one-way random |  | Avg. Agreement |  | ICC1k |  | 0.576 |  | 0.379 |  | 0.710 |  |
| two-way random |  | Avg. Agreement |  | ICC2k |  | 0.587 |  | 0.397 |  | 0.717 |  |
| two-way fixed |  | Avg. Consistency |  | ICC3k |  | 0.602 |  | 0.417 |  | 0.729 |  |
|  | | | | | | | | | | | |

**Plot Reliability Data**


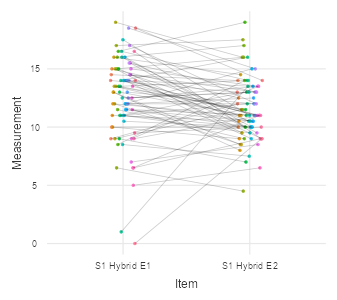


**Reliability Analysis**

Coefficient of Variation (%): 17.09Standard Error of Measurement (SEM): 2.1372Standard Error of the Estimate (SEE): 2.2948Standard Error of Prediction (SEP): 3.8012

| Intraclass Correlation Coefficients | | | | | | | | | | | |
| --- | --- | --- | --- | --- | --- | --- | --- | --- | --- | --- | --- |
| **Model** | | **Measures** | | **Type** | | **ICC** | | **Lower C.I.** | | **Upper C.I.** | |
| one-way random |  | Agreement |  | ICC1 |  | 0.570 |  | 0.428 |  | 0.685 |  |
| two-way random |  | Agreement |  | ICC2 |  | 0.571 |  | 0.429 |  | 0.685 |  |
| two-way fixed |  | Consistency |  | ICC3 |  | 0.573 |  | 0.431 |  | 0.688 |  |
| one-way random |  | Avg. Agreement |  | ICC1k |  | 0.726 |  | 0.599 |  | 0.813 |  |
| two-way random |  | Avg. Agreement |  | ICC2k |  | 0.727 |  | 0.601 |  | 0.813 |  |
| two-way fixed |  | Avg. Consistency |  | ICC3k |  | 0.729 |  | 0.603 |  | 0.815 |  |
|  | | | | | | | | | | | |

**Plot Reliability Data**


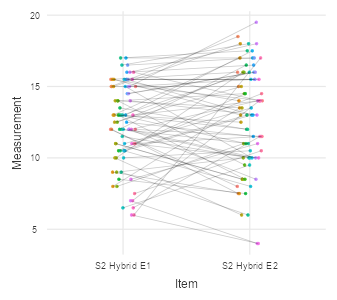


**Reliability Analysis**

Coefficient of Variation (%): 24.55Standard Error of Measurement (SEM): 1.4771Standard Error of the Estimate (SEE): 1.3705Standard Error of Prediction (SEP): 2.5118

| Intraclass Correlation Coefficients | | | | | | | | | | | |
| --- | --- | --- | --- | --- | --- | --- | --- | --- | --- | --- | --- |
| **Model** | | **Measures** | | **Type** | | **ICC** | | **Lower C.I.** | | **Upper C.I.** | |
| one-way random |  | Agreement |  | ICC1 |  | 0.405 |  | 0.288 |  | 0.511 |  |
| two-way random |  | Agreement |  | ICC2 |  | 0.413 |  | 0.296 |  | 0.518 |  |
| two-way fixed |  | Consistency |  | ICC3 |  | 0.424 |  | 0.308 |  | 0.528 |  |
| one-way random |  | Avg. Agreement |  | ICC1k |  | 0.577 |  | 0.447 |  | 0.676 |  |
| two-way random |  | Avg. Agreement |  | ICC2k |  | 0.585 |  | 0.456 |  | 0.682 |  |
| two-way fixed |  | Avg. Consistency |  | ICC3k |  | 0.595 |  | 0.471 |  | 0.691 |  |
|  | | | | | | | | | | | |

**Plot Reliability Data**


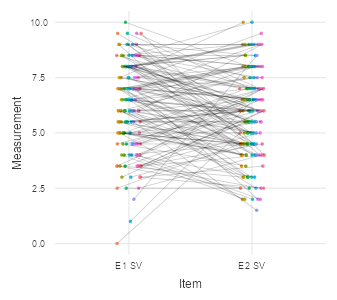


**Reliability Analysis**

Coefficient of Variation (%): 19.2Standard Error of Measurement (SEM): 1.1969Standard Error of the Estimate (SEE): 1.144Standard Error of Prediction (SEP): 2.0463

| Intraclass Correlation Coefficients | | | | | | | | | | | |
| --- | --- | --- | --- | --- | --- | --- | --- | --- | --- | --- | --- |
| **Model** | | **Measures** | | **Type** | | **ICC** | | **Lower C.I.** | | **Upper C.I.** | |
| one-way random |  | Agreement |  | ICC1 |  | 0.450 |  | 0.338 |  | 0.551 |  |
| two-way random |  | Agreement |  | ICC2 |  | 0.452 |  | 0.340 |  | 0.552 |  |
| two-way fixed |  | Consistency |  | ICC3 |  | 0.455 |  | 0.342 |  | 0.554 |  |
| one-way random |  | Avg. Agreement |  | ICC1k |  | 0.621 |  | 0.505 |  | 0.710 |  |
| two-way random |  | Avg. Agreement |  | ICC2k |  | 0.623 |  | 0.507 |  | 0.711 |  |
| two-way fixed |  | Avg. Consistency |  | ICC3k |  | 0.625 |  | 0.510 |  | 0.713 |  |
|  | | | | | | | | | | | |

**Plot Reliability Data**


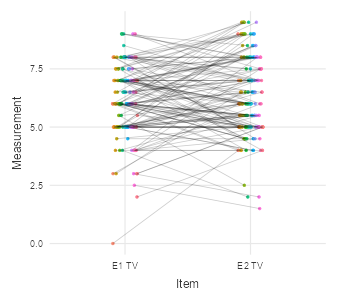


**Reliability Analysis**

Coefficient of Variation (%): 18.88Standard Error of Measurement (SEM): 2.3049Standard Error of the Estimate (SEE): 2.2995Standard Error of Prediction (SEP): 3.9913

| Intraclass Correlation Coefficients | | | | | | | | | | | |
| --- | --- | --- | --- | --- | --- | --- | --- | --- | --- | --- | --- |
| **Model** | | **Measures** | | **Type** | | **ICC** | | **Lower C.I.** | | **Upper C.I.** | |
| one-way random |  | Agreement |  | ICC1 |  | 0.497 |  | 0.389 |  | 0.591 |  |
| two-way random |  | Agreement |  | ICC2 |  | 0.497 |  | 0.389 |  | 0.591 |  |
| two-way fixed |  | Consistency |  | ICC3 |  | 0.497 |  | 0.389 |  | 0.591 |  |
| one-way random |  | Avg. Agreement |  | ICC1k |  | 0.664 |  | 0.561 |  | 0.743 |  |
| two-way random |  | Avg. Agreement |  | ICC2k |  | 0.664 |  | 0.561 |  | 0.743 |  |
| two-way fixed |  | Avg. Consistency |  | ICC3k |  | 0.664 |  | 0.560 |  | 0.743 |  |
|  | | | | | | | | | | | |

**Plot Reliability Data**


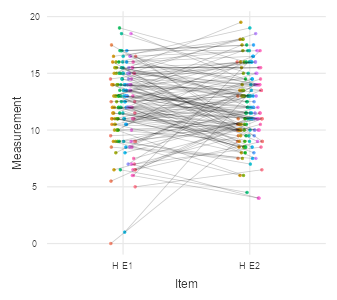


**Paired Samples T-Test**

| Paired Samples T-Test | | | | | | | | | | | |
| --- | --- | --- | --- | --- | --- | --- | --- | --- | --- | --- | --- |
|  | |  | |  | | **statistic** | | **df** | | **p** | |
| S1 E1 SV |  | S1 E2 SV |  | Student's t |  | 2.185 |  | 75.0 |  | 0.032 |  |
| S1 E1 TV |  | S1 E2 TV |  | Student's t |  | 1.240 |  | 75.0 |  | 0.219 |  |
| S1 Hybrid E1 |  | S1 Hybrid E2 |  | Student's t |  | 2.453 |  | 75.0 |  | 0.016 |  |
| S2 E1 SV |  | S2 E2 SV |  | Student's t |  | 1.795 |  | 75.0 |  | 0.077 |  |
| S2 E1 TV |  | S2 E2 TV |  | Student's t |  | -3.894 |  | 74.0 |  | < .001 |  |
| S2 Hybrid E1 |  | S2 Hybrid E2 |  | Student's t |  | -1.347 |  | 75.0 |  | 0.182 |  |
| E1 SV |  | E2 SV |  | Student's t |  | 2.834 |  | 151.0 |  | 0.005 |  |
| E1 TV |  | E2 TV |  | Student's t |  | -1.542 |  | 149.0 |  | 0.125 |  |
| H E1 |  | H E2 |  | Student's t |  | 0.646 |  | 151.0 |  | 0.519 |  |
| Note. Hₐ μ _Measure 1 - Measure 2_ ≠ 0 | | | | | | | | | | | |
|  | | | | | | | | | | | |

**Paired Samples T-Test**

| Paired Samples T-Test | | | | | | | | | | | |
| --- | --- | --- | --- | --- | --- | --- | --- | --- | --- | --- | --- |
|  | |  | |  | | **Statistic** | | **df** | | **p** | |
| S1 E1 SV |  | S1 E2 SV |  | Student's t |  | 2.185 |  | 75.0 |  | 0.032 |  |
|  |  |  |  | Wilcoxon W |  | 1692 | ᵃ |  | | 0.034 |  |
| S1 E1 TV |  | S1 E2 TV |  | Student's t |  | 1.240 |  | 75.0 |  | 0.219 |  |
|  |  |  |  | Wilcoxon W |  | 1456 | ᵇ |  | | 0.047 |  |
| S1 Hybrid E1 |  | S1 Hybrid E2 |  | Student's t |  | 2.453 |  | 75.0 |  | 0.016 |  |
|  |  |  |  | Wilcoxon W |  | 1955 | ᵈ |  | | 0.002 |  |
| S2 E1 SV |  | S2 E2 SV |  | Student's t |  | 1.795 |  | 75.0 |  | 0.077 |  |
|  |  |  |  | Wilcoxon W |  | 1555 | ᵉ |  | | 0.067 |  |
| S2 E1 TV |  | S2 E2 TV |  | Student's t |  | -3.894 |  | 74.0 |  | < .001 |  |
|  |  |  |  | Wilcoxon W |  | 617 | ᵉ |  | | < .001 |  |
| S2 Hybrid E1 |  | S2 Hybrid E2 |  | Student's t |  | -1.347 |  | 75.0 |  | 0.182 |  |
|  |  |  |  | Wilcoxon W |  | 1097 | ᶠ |  | | 0.300 |  |
| E1 SV |  | E2 SV |  | Student's t |  | 2.834 |  | 151.0 |  | 0.005 |  |
|  |  |  |  | Wilcoxon W |  | 6454 | ᵍ |  | | 0.005 |  |
| E1 TV |  | E2 TV |  | Student's t |  | -1.542 |  | 149.0 |  | 0.125 |  |
|  |  |  |  | Wilcoxon W |  | 4035 | ʰ |  | | 0.220 |  |
| H E1 |  | H E2 |  | Student's t |  | 0.646 |  | 151.0 |  | 0.519 |  |
|  |  |  |  | Wilcoxon W |  | 5924 | ⁱ |  | | 0.213 |  |
| Note. Hₐ μ _Measure 1 - Measure 2_ ≠ 0 | | | | | | | | | | | |
| ᵃ 4 pair(s) of values were tied | | | | | | | | | | | |
| ᵇ 9 pair(s) of values were tied | | | | | | | | | | | |
| ᵈ 2 pair(s) of values were tied | | | | | | | | | | | |
| ᵉ 6 pair(s) of values were tied | | | | | | | | | | | |
| ᶠ 5 pair(s) of values were tied | | | | | | | | | | | |
| ᵍ 10 pair(s) of values were tied | | | | | | | | | | | |
| ʰ 15 pair(s) of values were tied | | | | | | | | | | | |
| ⁱ 7 pair(s) of values were tied | | | | | | | | | | | |
|  | | | | | | | | | | | |
