# Supplementary material for: Enhancing medical assessment strategies: a comparative study between structured, traditional and hybrid viva-voce assessment
Source: BMC Med Educ. 2025 Jun 4;25:835. doi: 10.1186/s12909-025-07428-9 (PMC12139079; doi:10.1186/s12909-025-07428-9)
Supplement: Supplementary file 3 — Supplementary Material 3 [file 12909_2025_7428_MOESM3_ESM.pdf]

Biochemistry Structured viva sessional III

https://forms.gle/76JkZUB8f2TWMeuy8

1. Reference values of (1)
    1. Urea
    2. Fasting glucose
2. Biochemical defect in(1 mark)
    1. Phenylketonuria
    2. Von Gierkes disease
3. Mechanism of action of any aspirin(1)
4. Diagnostic significance of HbA1c (1)
5. Describe the functions of NAD(1)
6. Give reasons for ketoacidosis as a complication in type 1 diabetes mellites(1)
7. Justify Lysosome is involved in autophagy.(2)
8. Justify: pyridoxine deficiency causes anemia (2)

1. **Reference values of (1)**
    1. **HbA1c**
    2. **Creatinine(adult male)**
2. **Biochemical defect in(1 mark)**
    - **Porphyria cutanea tarda**
    - **Hereditary fructose intolerance**
3. **Mechanism of action of allopurinol drug(1)**
4. **Diagnostic significance of Bence Jones protein in urine(1)**
5. **Describe the functions of S adenosyl methionine(1)**
6. **Give reasons ascites in cases of hypoproteinemia(1)**
7. **Justify Trans fatty acid is atherogenic.(2)**
8. **Justify hyperkalemia is seen in patients with diabetic ketoacidosis. (2)**

1. **Reference values of (1)**
    1. **Serum Creatinine of adult female**
    2. **Fasting blood glucose**
2. **Biochemical defect in(1 mark)**
    1. **Pompe's Disease**
    2. **Hartnup's disease**
3. **Mechanism of action of dicoumarol (1)**
4. **Diagnostic significance of Troponin I (1)**
5. **Describe the functions of Heme(1)**
6. **Give reasons for anemia in pyridoxin deficiency(1)**
7. **Justify Ketone bodies can be synthesized by liver but cannot be utilized by liver..(2)**
8. **Justify thiamine deficiency can cause lactic acidosis. (2)**

1. Reference values of (1)
    1. Serum HDL
    2. Serum albumin
2. Biochemical defect in(1 mark)
    1. Gaucher disease
    2. Maple Syrup urine disease
3. Mechanism of action of penicillin drug(1)
4. Diagnostic significance of Ca125 (1)
5. Describe the functions of creatine(1)
6. Give reasons for hyperbilirubinemia in cholelithiasis (1)
7. Justify Hyperammonemia causes brain toxicity. .(2)
8. Justify: pyridoxine deficiency causes neuronal symptoms. (2)

1. Reference values of (1)
   1. Serum total protein
   2. Random blood glucose
2. Biochemical defect in(1 mark)
   1. Acute intermittent porphyria
   2. Steatorrhea
3. Mechanism of action of glycine in hyperammonaemia (1)
4. Diagnostic significance of prostate specific antigen (1)
5. Describe the functions of epinephrine(1)
6. Give reasons for ascites in kwashiorkor (1)
7. Justify Hepatic encephalopathy is seen in liver cirrhosis.(2)
8. Justify: vitamin A deficiency causes nyctalopia. (2)

1. **Reference values of (1)**
    1. **Serum uric acid**
    2. **Serum sodium**
2. **Biochemical defect in(1 mark)**
    1. **Albinism**
    2. **Lesch-Nyhan Syndrome**
3. **Mechanism of action of nitro-glycerine (1)**
4. **Diagnostic significance of gamma glutamyl transferase(GGT) (1)**
5. **Describe the functions of thyroxine(1)**
6. **Give reasons lactic acidosis in hypoxia(1)**
7. **Justify Chronic alcoholics may present with hypoglycemia.(2)**
8. **Justify calcium deficiency causes tetany. (2)**

1. Reference values of (1)
    1. Serum LDL
    2. Serum HDL
2. Biochemical defect in(1 mark)
    1. Alkaptonuria
    2. scurvy
3. Mechanism of action of sildenafil citrate (1)
4. Diagnostic significance of OGCT(oral glucose challenge test)(1)
5. Describe the functions of tetrahydrobiopterin(1)
6. Give reasons for respiratory distress syndrome in premature newborn.(1)
7. JustifyEarly diagnosis and treatment of phenylketonuria prevents occurrence of mental retardation. (2)
8. Justify vitamin B12 deficiency causes peripheral neuropathy. [illegible]

1. **Reference values of (1)**
    1. **Serum phosphate**
    2. **Serum Creatinine**
2. **Biochemical defect in(1 mark)**
    1. **Phenylketonuria**
    2. **Pellagra**
3. **Mechanism of action of aspirin(1)**
4. **Diagnostic significance of fat in stool (1)**
5. **Describe the functions of cytochrome oxidase(1)**
6. **Give reasons for neuropsychiatric symptoms in acute intermittent porphyria (1)**
7. **Justify Oxidized LDL is atherogenic .(2)**
8. **Justify: hypoalbuminemia patients also have hypocalcemia . (2)**

1. **Reference values of (1)**
    1. **Serum Alkaline phosphatase**
    2. **Serum potassium**
2. **Biochemical defect in(1 mark)**
    1. **Gaucher disease**
    2. **Nyctalopia**
3. **Mechanism of action of allopurinol drug(1)**
4. **Diagnostic significance of TSH (1)**
5. **Describe the functions of ALA synthase(1)**
6. **Give reasons for skin damage in porphyria cutanea tarda(1)**
7. **Justify Arachidonic acid is not essential fatty acid.(2)**
8. **Justify premature newborn is more prone to develop kernicterus . (2)**

1. **Reference values of (1)**
   1. **Post prandial blood glucose**
   2. **Serum Total cholesterol**
2. **Biochemical defect in(1 mark)**
   1. **Essential fructosuria**
   2. **Homocystinuria- Type I**
3. **Mechanism of action of methotrexate drug(1)**
4. **Diagnostic significance of Cortisol (1)**
5. **Describe the functions of Nitric oxide (1)**
6. **Give reasons frequency of micturition in diabetes mellites (1)**
7. **Justify Prolonged cortisone therapy leads to minerals imbalance .(2)**
8. **Justify vitamin D deficiency causes rickets. (2)**

1. **Reference values of (1)**
    1. **Serum sodium**
    2. **HCO3**
2. **Biochemical defect in(1 mark)**
    1. **Hyperammonemia-Type I**
    2. **Respiratory Distress Syndrome in newborn**
3. **Mechanism of action of dicoumarol (1)**
4. **Diagnostic significance of ferric chloride test in urine (1)**
5. **Describe the functions of glutathione (1)**
6. **Give reasons for cataract in diabetes mellites (1)**
7. **Justify primary hypothyroidism has elevated TSH levels (2)**
8. **Justify vitamin A deficiency causes keratomalacia . (2)**

1. **Reference values of (1)**
    1. **Urea**
    2. **Fasting glucose**

2. **Biochemical defect in(1 mark)**
    1. **Niemann pick disease**
    2. **Tetany**
3. **Mechanism of action of sulfonamide (1)**
4. **Diagnostic significance of Vitamin D levels (1)**
5. **Describe the functions of phenylalanine hydroxylase(1)**
6. **Give reasons pedal edema in patients with renal failure(1)**
7. **Justify Pyridoxine deficiency causes peripheral neuropathy (2)**
8. **Justify classical galactosemia causes cataract. (2)**

1. **Reference values of (1)**
   1. **Serum total protein**
   2. **Serum Creatinine in adult male**
2. **Biochemical defect in(1 mark)**
   1. **Niemann pick disease**
   2. **Multiple myeloma**
3. **Mechanism of action of penicillin (1)**
4. **Diagnostic significance of CK-MB(1)**
5. **Describe the functions of phosphofructokinse(1)**
6. **Give reasons for hemolysis in patients with sickle cell anemia(1)**
7. **Justify Hepatic encephalopathy is seen in liver cirrhosis.(2)**
8. **Justify diabetes mellites patients are more prone to develop cataract. (2)**

1. **Reference values of (1)**
    1. **Blood pH**
    2. **Blood PCO2**
2. **Biochemical defect in(1 mark)**
    1. **Lactose intolerance**
    2. **Galactosemia**
3. **Mechanism of action of 5 fluorouracil (1)**
4. **Diagnostic significance of adenosine deaminase (ADA) (1)**
5. **Describe the functions of insulin(1)**
6. **Give reasons for anemia in G-6PD deficiency(1)**
7. **Justify: Urea levels are elevated during starvation.(2)**
8. **Justify: central Indian population has higher incidence of pellagra . (2)**

1. **Reference values of (1)**
    1. **Serum calcium**
    2. **Random blood glucose**
2. **Biochemical defect in(1 mark)**
    1. **Burning feet syndrome**
    2. **Pheochromocytoma**
3. **Mechanism of action of sulfonamide (1)**
4. **Diagnostic significance of alkaline phosphatase (1)**
5. **Describe the functions of glucagon(1)**
6. **Give reasons for elevation of amylase levels in pancreatitis(1)**
7. **Justify Digestive enzymes are secreted as zymogens.(2)**
8. **Justify folic acid is supplemented in pregnant women. (2)**

1. **Reference values of (1)**
    1. **Post prandial blood glucose**
    2. **Serum Total cholesterol**
2. **Biochemical defect in(1 mark)**
    1. **Gilberts syndrome**
    2. **Hartnups disease**
3. **Mechanism of action of methotrexate (1)**
4. **Diagnostic significance of troponin T(1)**
5. **Describe the functions of cholesterol (1)**
6. **Give reasons cataract is seen in patients with classical galactosemia(1)**
7. **Justify: oxidised LDL is atherogenic(2)**
8. **Justify: hyperbilirubinemia is seen in patients with hemolysis. (2)**

1. **Reference values of (1)**
    1. **Serum Alkaline phosphatase**
    2. **Serum calcium**
2. **Biochemical defect in(1 mark)**
    1. **Lactose intolerance**
    2. **Respiratory distress syndrome in newborn**
3. **Mechanism of action of 5 fluorouracil (1)**
4. **Diagnostic significance of VMA in urine (1)**
5. **Describe the functions of melanin(1)**
6. **Give reasons for conjugated hyperbilirubinemia in cases of obstructive jaundice (1)**
7. **Justify-prolonged starvation leads to ketoacidosis(2)**
8. **Justify- diabetic patients are advised against consuming foods with high glycemic index. (2)**

1. **Reference values of (1)**
   1. **Serum HDL**
   2. **Serum albumin**
2. **Biochemical defect in(1 mark)**
   1. **Galactosemia**
   2. **Refsums disease**
3. **Mechanism of action of statin (1)**
4. **Diagnostic significance of 5-HIAA in urine (1)**
5. **Describe the functions of carnitine (1)**
6. **Give rationale for use of oral rehydration (ORS) therapy on dehydration (1)**
7. **Justify Early diagnosis and treatment of phenylketonuria prevents occurrence of mental retardation.(2)**
8. **Justify high fiber diet in considered good for health. (2)**

1. Reference values of (1)
    1. Serum uric acid
    2. Serum sodium
2. Biochemical defect in(1 mark)
    1. Pernicious anemia
    2. Lesch nyhans syndrome
3. Mechanism of action of glycine in hyperammonemia (1)
4. Diagnostic significance of lipase(1)
5. Describe the functions of histidine (1)
6. Give reasons skin sensitivity in porphyria cutanea tarda (1)
7. Justify-Glycerol cannot be converted to glycerol 3 phosphate in adipose tissue.(2)
8. Justify- vitamin E has anti-oxidant properties (2)

1. **Reference values of (1)**
   1. **HbA1c**
   2. **Creatinine(adult male)**
2. **Biochemical defect in(1 mark)**
   1. **Hurtnups disease**
   2. **Von Geirkes disease**
3. **Mechanism of action of nitroglycerine drug(1)**
4. **Diagnostic significance of amylase(1)**
5. **Describe the functions of LCAT(1)**
6. **Give reasons for hyperuricemia in Von Geirk's disease (1)**
7. **JustifyKetone bodies can be synthesized by liver but cannot be utilized by liver.(2)**
8. **Justify vitamin B12 is always supplemented with folic acid . (2)**

1. **Reference values of (1)**
    1. **Serum Alkaline phosphatase**
    2. **Serum uric acid**
2. **Biochemical defect in(1 mark)**
    1. **Gaucher’s disease**
    2. **Albinism**
3. **Mechanism of action of sildenafil citrate (1)**
4. **Diagnostic significance of acid phosphatase(1)**
5. **Describe the functions of PAPS(1)**
6. **Give reasons for any clinical feature of any disease (1)**
7. **Justify Oxidized LDL is atherogenic .(2)**
8. **Justify copper deficiency causes anemia. (2)**
